# The impact of high-glucose or high-fat diets on the metabolomic profiling of mice

**DOI:** 10.3389/fnut.2023.1171806

**Published:** 2023-07-10

**Authors:** Dadi Xie, Yanbo Zhang, Yujin Guo, Xianzhong Xue, Shiyuan Zhao, Chunmei Geng, Yuanyuan Li, Rui Yang, Yizhang Gan, Hanbing Li, Zhongfa Ren, Pei Jiang

**Affiliations:** ^1^Department of Endocrinology, Tengzhou Central People’s Hospital, Tengzhou, China; ^2^Xuzhou Medical University, Xuzhou, China; ^3^Jining First People’s Hospital, Jining Medical University, Jining, China; ^4^Department of Paediatrics, Tengzhou Central People’s Hospital, Tengzhou, China

**Keywords:** diet, glucolipid, tissue, gas chromatography-mass spectrometry, metabolome

## Abstract

**Objective:**

Diets high in glucose or fat contribute to an increased prevalence of the diseases. Therefore, the objective of the current research was to observe and evaluate the impact of dietary components on different metabolomic profiles in primary tissues of mice.

**Methods:**

For 8 weeks, diet with high-glucose or-fat was given to C57BL/6 J mice. The levels of metabolites in the primary tissues of mice were studied using gas chromatography-mass spectrometry (GC-MS) and analyzed using multivariate statistics.

**Results:**

By comparing the metabolic profiles between the two diet groups and control group in mice main tissues, our study revealed 32 metabolites in the high-glucose diet (HGD) group and 28 metabolites in the high-fat diet (HFD) group. The most significantly altered metabolites were amino acids (AAs; L-alanine, L-valine, glycine, L-aspartic acid, L-isoleucine, L-leucine, L-threonine, L-glutamic acid, phenylalanine, tyrosine, serine, proline, and lysine), fatty acids (FAs; propanoic acid, 9,12-octadecadienoic acid, pentadecanoic acid, hexanoic acid, and myristic acid), and organic compounds (succinic acid, malic acid, citric acid, L-(+)-lactic acid, myo-inositol, and urea). These metabolites are implicated in many metabolic pathways related to energy, AAs, and lipids metabolism.

**Conclusion:**

We systematically analyzed the metabolic changes underlying high-glucose or high-fat diet. The two divergent diets induced patent changes in AA and lipid metabolism in the main tissues, and helped identify metabolic pathways in a mouse model.

## Introduction

High-energy food intake is a characteristic of the Western-style diet (1). Dietary intake patterns are linked with glucolipid metabolic disorders which are a series of diseases associated with the metabolic disturbance of glucose and lipids (2). Glucolipid metabolic disorders can lead to many health problems such as obesity, diabetes, hypertension, coronary heart disease, and steatohepatitis (2). The incidence of obesity and diabetes has also risen in younger individuals with morbidity significantly increasing in children and young people (3, 4). To mitigate these diseases, dietary education is usually recommended. Studies on glucolipid metabolism investigating metabolic disorders in target tissues support findings of how dietary changes influence health.

Metabolomic has played a critical role in assessing and diagnosing health and disease of individuals; it allows for a thorough examination of low-molecular-weight molecules in biological samples (5). Multiple metabolomic techniques such as gas chromatography–mass spectrometry (GC-MS) and liquid-chromatography mass spectrometry (LC-MS), have been employed to uncover the specificity and complexity of metabolic alterations within animal and human tissues (6). The goal of metabolomics is to develop diagnostic and mechanistic biochemical biomarkers that can monitor changes in the metabolic homeostasis of individuals (7). This requires the identification of specific metabolites that play a role in health and disease. In the field of nutritional metabolomics, small molecule chemical profiling is used to promote dietary information integration to forecast health and complicated pathobiology, complex biosystems research, and underline the essential role of nutrition and food in health and disease integrated biosystem models (8).

Diets have long been suspected by researchers to have a role in the development of obesity and other metabolic diseases, however, in the last few decades they have been the focus of a comprehensive investigation. To date, most studies that have revealed the intimate interplay between diet style and metabolism have been performed on a limited number of samples or tissues in animal models (9, 10). There are few studies to evaluate the metabonomic changes of the main tissues in animal models comprehensively. Additionally, there is currently a lack of data comparing the effects of the glucose or fat diet on the metabolism of main tissues. Therefore, in order to evaluate the role of diet in metabolomic variations of the main tissues in animal models comprehensively. The experimental design of this work is the first to compare the metabolomic responses of the main tissues in mice fed with different dietary glucose and fat levels by using the GC/MS-based metabolomics approach. Consequently, the results of this study enhance our understanding of the metabolic alterations that occur in mice under an obesogenic environment and exploring optimal nutritional requirements and feeding regimes.

## Materials and methods

### Experimental animal and dietary intrusion

Eventually, the acquired 8-week-old male C57BL/6 J mice were kept in a colony room providing a 12/12 h light/dark cycle at 22–23°C, respectively. Before being utilized for experiments, all mice were given 7 days to acclimate. Following that, the mice were categorized randomly into three groups: control group, high-glucose diet (HGD) group, high-fat diet (HFD) group, and each group with 7 mice (*n* = 7 per group). The composition of diet provided to each group as Ain-93 M (control); 75.9% carbohydrate, 14.7% protein and 9.4% fat (high-glucose diet); 25% carbohydrate, 15% protein, and 60% fat (high-fat diet) at Jiangsu Synergy Pharmaceutical & Biological Engineering Co., Nanjing, CHA (Supplementary file 1). For 8 weeks, all diets were given *ad libitum*. Mice were weighed weekly and their food and drink intake were assessed twice a week. According to national rules and with the agreement of Jining Medical University’s ethics committee (Protocol #JNMC2020DWRM0076), animal studies were conducted.

### Tissue sampling

Following a 12-h overnight fast, mice were euthanized with 1% sodium pentobarbital (50 mg/kg). Subsequently, the tail artery was punctured via needle to evaluate total cholesterol (TC), triglycerides (TG), and glucose levels, from the pricked blood sample. A portable glucometer was used to monitor blood glucose levels (ACCU109 CHEK, Roche, IN). Wako Inc. (Richmond, VA, United States) kits were used to quantify TG and TC. To isolate serum, the blood was immediately drawn and centrifuged at 5,000 rpm for 6 min. Furthermore, the mice were slaughtered by dislocation of the cervical. On an ice surface, the mice were instantly dissected. 0.9% physiological saline was used to cleanse the whole heart, liver, brain, and kidney samples before freezing them in liquid nitrogen and preserving them in storage at −80°C for future use.

### Sample pretreatment for GC-MS

Serum Samples Preparation: The 350 μL methanol (having 100 μg/mL Internal Standard Heptadecanoic acid, 98% purity; lot: SLBX4162) was mixed with 100 μL serum. The prepared mixture was then centrifuged at 4°C at 14,000 rpm for 10 min. Later, the obtained supernatant was transferred to a 2-mL tube and dried under nitrogen gas at 37°C. The extracts were then mixed with 80 μL of O-methyl hydroxylamine hydrochloride (purity: 98.0%; lot: LG10T16, 15 mg/mL in pyridine; J&K Scientific Ltd. Beijing, CHA) and incubated at 70 °C for 90 min. After that, each sample was subjected to 100 μL N, O-bis-(trimethylsilyl) trifluoroacetamide with 1% trimethylchlorosilane (BSTFA +1% TMCS, v/v; lot: BCBZ4865, Sigma-Aldrich, MO, United States), incubated for 60 min at 70°C. Before GC–MS analysis, the prepared samples were vortexed, centrifuged at 4°C for 2 min at 14,000 rpm, and filtered via a 0.22-μm filter membrane.

Tissue samples: For this experiment, methanol (Thermo Fisher Scientific, Waltham, MA, United States) was used to homogenize the 50 mg of tissue (kidney, liver, heart, and brain), transferred to a 2-mL tube with 50 μL 1 mg/mL IS. Mixtures were further centrifuged for 10 min at 14,000 rpm and 4°C to isolate the solid particles. The remaining procedure was similar to that of serum samples.

### GC-MS analysis

A 7890B gas chromatograph (GC) machine was utilized in conjunction with a 7000C mass spectrometer (MS) and an HP-5MS fused silica capillary column (Agilent Technologies, CA, United States) for GC-MS analysis. Each 1 μL aliquot of the derivatized solution was processed in splitting operation at a flow rate of 1 mL/min of helium gas through the column (50: 1). The GC temperature protocol started at 60°C for 4 min, then raised by 8°C/min to 300°C for 5 min. The temperatures of the injection, transfer line, and ion source were 280°C, 250°C, and 230°C, respectively. Electrospray ionization was used to record 20 scans per second across 50–800 m/z.

### Multivariate analysis

Weight, blood glucose, TG, and TC levels were compared using a two-way ANOVA. For all measurements, the mean ± standard error of the mean was used. Significantly, the obtained was visualized and interpreted via GraphPad Prism 8 (GraphPad Software Inc., La Jolla, CA, United States) with applied a *p*-value < 0.05.

### Data processing and metabolite identification

Analysis of unknown preprocessing of GC data was performed using MassHunter (Agilent Technologies, CA, USA). The raw data were converted to the m/z data format. We created a library containing all QC samples, and the NIST (U.S. National Institute of Standards and Technology) 14 GC-MS library was used to identify the unknown metabolites from QC library. Further, the data were analyzed by alignment, retention time correction, baseline filtration, and deconvolution. Then, the metabolites with similarity >80% were considered as structurally identified. Afterwards, a new spectrum library named “New Library” was obtained, and the metabolites of samples were identified using this New Library. Finally, an integrated data matrix composed of the peak index (RT-m/z pair), sample name, and corresponding peak area was generated. Subsequently, the peak area from the data matrix was normalized using Microsoft Excel.

The statistical analyses were performed using **SIMCA-P v14.0** (Umetrics, Umea, Sweden). Additionally, orthogonal projection to latent structures discriminant analysis (**OPLS-DA**) was used to distinguish amongst the control, HGD, and HFD group with applied significant cutoff value as a *p*-value and variable importance in projection (VIP) values set as <0.05 and >1.0, respectively. Moreover, using permutation testing, the HGD and HFD groups were given one last look-over (200 permutations). We conducted our statistical analyses using **SPSS** Inc.’s (Chicago, IL) Statistical Package for the Social Sciences (19.0). To conduct functional analyses, **MetaboAnalyst v5.0** and the Kyoto Encyclopedia of Genes and Genomes (**KEGG**^1^) were utilized. A raw *p*-value < 0.05 was classified as significant, and an impact value >0 was considered to be significant. In earlier research, several standard metabolomic analysis methodologies were used (11).

## Results

### Metabolic changes induced by HGD and HFD

At the start of the trial, the average body weight was 18.94 ± 0.34 g (Table 1). Compared to the beginning of the experiment, each group’s animals gained weight by the study’s end, the control group gained 3.54 ± 0.82 g, the HGD group gained 6.83 ± 0.79 g, the HFD group gained 9.43 ± 0.41 g. Mice in the HGD and HFD diet groups gained considerably more weight than mice in the control group after 8 weeks on the diets (*p* < 0.05). Furthermore, the weight of the HFD group was increased more obviously compared with the HGD group (*p* < 0.05). The fasting blood glucose concentration was increased significantly in the HGD and HFD groups (*p* < 0.01) than the control group, as well as the TG and TC (*p* < 0.05). And the glucose, TG and TC increased in the HFD group as compared to that of the HGD group (*p* < 0.01).

**Table 1:** The dietary effect on body weight and serum biochemical parameters.

Group		Initial weight (g)	Final weight (g)	Glucose (mg/dl)	TG (mg/dl)	TC (mg/dl)
CON	Mean ± SD	18.93 ± 0.35	22.47 ± 1.17	99.07 ± 2.71	32.0 ± 2.09	53.26 ± 4.24
HGD	Mean ± SD	18.80 ± 0.36	25.63 ± 1.15	164.43 ± 4.39	61.20 ± 4.39	70.13 ± 6.81
	Versus CON	1.000	0.022*	0.000*	0.000*	0.015*
	Versus HFD	1.000	0.032*	0.000*	0.003*	0.006*
HFD	Mean ± SD	19.10 ± 0.30	28.53 ± 0.71	189.10 ± 3.60	79.40 ± 3.31	90.93 ± 3.56
	Versus CON	1.000	0.001*	0.000*	0.000*	0.000*
	Versus HGD	1.000	0.032*	0.000*	0.003*	0.006*

TC, total cholesterol; TG, total triglyceride. Values are means ± SD, *n* = 7, *p-value significant.

### Serum and tissue sample GC-MS total ion chromatograms

Consequently, representative GC–MS TICs of quality control (QC) serum and tissue samples (liver, heart, kidney and brain) from control groups and HGD mixture, and control groups and HFD mixture exhibited robust signals and high RT repeatability. The details of TICs among different tissues could be observed in Supplementary file 2.

### Metabolomics analyses of tissue sample and serum

**Figure 1:**
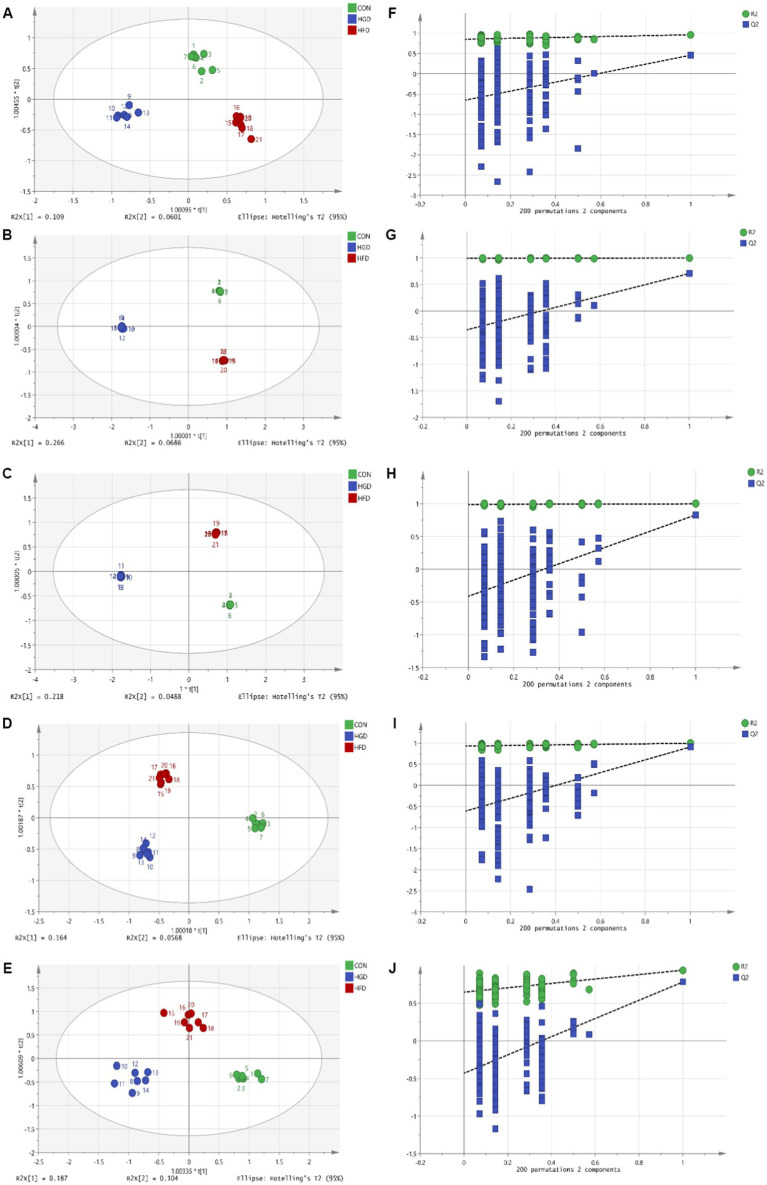
OPLS-DA scores (**A**: serum, **B**: heart, **C**: liver, **D**: brain, **E**: kidney) and 200 permutation tests (**F**: serum, **G**: heart, **H**: liver, **I**: brain, **J**: kidney) for two diet groups and a control group in the OPLS-DA models.

The parameters obtained from the OPLS-DA showed efficient model that evidently separated the control and two diet groups (serum: R2X = 0.671, R2Y = 0.968, Q2 = 0.573; heart: R2X = 0.867, R2Y = 1, Q2 = 0.826; liver: R2X = 0.907, R2Y = 1, Q2 = 0.877; brain: R2X = 0.822, R2Y = 0.99, Q2 = 0.548; kidney: R2X = 0.692, R2Y = 0.95, Q2 = 0.78) (Figures 1A–E). Parameter values near to 1.0 imply a model that is stable and predictable. In Figures 1F–J, the blue Q2 points regression line crosses the vertical axis (on the left) below zero, indicating that the OPLS-DA models are not overfitting.

### Identification of metabolites

OPLS-DA with VIP and *p*-value of the *t*-test are the standard criteria for potential metabolites. VIP >1.0 and *p* < 0.05 in comparison to the control group indicated differences in the metabolites between the two diet groups. In addition, FC (Fold Change: HGD group/control group or HFD group/control group) >1 indicated that the metabolite has an upward trend, while FC < 1 indicated a downward trend. The **HGD** group alone changed 32 metabolites including AA derivatives, FAs, nucleosides and bases, TCA cycle, and other metabolites. There were 9 increased metabolites and 2 decreased metabolites in serum. In heart tissue, HGD led to 12 up-regulated altered metabolites. In liver tissue, 12 differential metabolites including 11 up-regulated and 1 down-regulated metabolites. In brain tissue, 10 up-regulated altered metabolites were identified. In addition, 7 up-regulated differential metabolites in kidney tissue. The **HFD** changed 28 metabolites including AA derivatives, FAs, and other metabolites. Ten metabolites (9 up-regulated and 1 down-regulated) in serum, 10 up-regulated in heart, 7 up-regulated metabolites in liver, 4 up-regulated metabolites in brain, 6 up-regulated metabolites in kidney were observed. Table 2 contains detailed metabolite findings.

**Table 2:** Metabolomics of target tissues in mice as a result of treatment with HGD and HFD.

	HGD				HFD			
Tissue	Metabolites	HMDB	VIP	Trend	Metabolites	HMDB	VIP	Trend
Serum	L-Alanine	HMDB0000161	2.23	↑	Lysine	HMDB0000182	2.52	↑
	Glutamate	HMDB0000148	3.04	↑	Glycine	HMDB0000123	1.59	↑
	Glycine	HMDB0000123	1.20	↑	Proline	HMDB0000162	1.58	↑
	L-Leucine	HMDB0000687	1.88	↑	L-Alanine	HMDB0000161	2.05	↑
	Tyrosine	HMDB0000158	2.08	↑	Tyrosine	HMDB0000158	1.81	↑
	L-Aspartic acid	HMDB0000191	1.45	↑	Glycerol	HMDB0000131	1.40	↑
	Propanoic acid	HMDB0000237	1.83	↓	Aminomalonic acid	HMDB0001147	1.12	↓
	Succinic acid	HMDB0000254	1.66	↑	Cholesterol	HMDB0000067	1.44	↑
	Myo-inositol	HMDB0000211	1.89	↑	Palmitate	HMDB0000220	1.48	↑
	d-Glucose	HMDB0000122	1.89	↑	Myo-inositol	HMDB0000211	1.59	↑
	Uric acid	HMDB0000289	1.36	↓				
Heart	L-Alanine	HMDB0000161	1.64	↑	d-Glucose	HMDB0000122	1.98	↑
	Hexanoic acid	HMDB0000535	1.48	↑	Inositol	HMDB0000211	2.12	↑
	L-Valine	HMDB0000883	1.65	↑	Adenosine	HMDB0000050	2.60	↑
	L-Isoleucine	HMDB0000172	1.55	↑	L-Alanine	HMDB0000161	1.46	↑
	Uracil	HMDB0000300	1.62	↑	Aspartic acid	HMDB0000191	1.10	↑
	Serine	HMDB0062263	1.48	↑	L-Threonine	HMDB0000167	1.78	↑
	Aspartic acid	HMDB0000191	1.52	↑	Hexane	HMDB0029600	1.06	↑
	L-Glutamic acid	HMDB0000148	1.62	↑	propanoic acid	HMDB0000237	1.21	↑
	Phenylalanine	HMDB0000159	1.42	↑	L-Glutamic acid	HMDB0000148	1.54	↑
	Tyrosine	HMDB0000158	1.08	↑				
	Myo-Inositol	HMDB0000211	1.37	↑				
	Adenosine	HMDB0000050	1.45	↑				
Liver	Alanine	HMDB0000161	1.13	↑	Valine	HMDB0000883	2.21	↑
	Acetamide	HMDB0031645	1.80	↓	4-Aminobutanoic acid	HMDB0000112	2.09	↑
	Succinic acid	HMDB0000254	1.55	↑	Myristic acid	HMDB0000806	2.32	↑
	L-Valine	HMDB0000883	1.33	↑	d-Proline	HMDB0003411	1.66	↑
	Urea	HMDB0000294	1.37	↑	Glycine	HMDB0000123	2.95	↑
	Glycine	HMDB0000123	1.37	↑	9,12-Octadecadienoic acid	HMDB0000673	2.87	↑
	Aspartic acid	HMDB0000191	1.21	↑	9-Octadecenamide	HMDB0002117	1.94	↑
	Pentadecanoic acid	HMDB0000826	1.15	↑				
	Galactinol	HMDB0005826	1.31	↑				
	D-Mannose	HMDB0000169	1.38	↑				
	D-Myo-Inositol	HMDB0000211	1.25	↑				
	Adenosine	HMDB0000050	1.16	↑				
Brain	Phosphoric acid	HMDB0002142	1.01	↑	L-Aspartic acid	HMDB0000191	2.44	↑
	Norleucine	HMDB0001645	1.83	↑	Uridine	HMDB0000296	1.45	↑
	L-Aspartic acid	HMDB0000191	1.57	↑	L-(+)-Lactic acid	HMDB0000190	1.37	↑
	Aminomalonic acid	HMDB0001147	1.55	↑	L-Alanine	HMDB0000161	1.39	↑
	Malic acid	HMDB0000744	1.21	↑				
	L-5-Oxoproline	HMDB0000267	1.09	↑				
	L-Phenylalanine	HMDB0000159	1.39	↑				
	Citric acid	HMDB0000094	1.12	↑				
	L-[+]-Lactic acid	HMDB0000190	1.21	↑				
	Phosphorylethanolamine	HMDB0000224	1.59	↑				
Kidney	Glycine	HMDB0000123	1.92	↑	L-Alanine	HMDB0000161	1.24	↑
	L-Leucine	HMDB0000687	1.13	↑	L-Leucine	HMDB0000687	1.53	↑
	L-Aspartic acid	HMDB0000191	1.40	↑	L-Valine	HMDB0000883	1.28	↑
	Tyrosine	HMDB0000158	1.56	↑	Pentanedioic acid	HMDB0000661	1.39	↑
	Glycerol	HMDB0000131	1.71	↑	Phosphoric acid	HMDB0002142	2.00	↑
	Xylitol	HMDB0002917	2.03	↑	Myo-Inositol	HMDB0000211	1.24	↑
	Phosphoric acid	HMDB0002142	1.26	↑				

**Figure 2:**
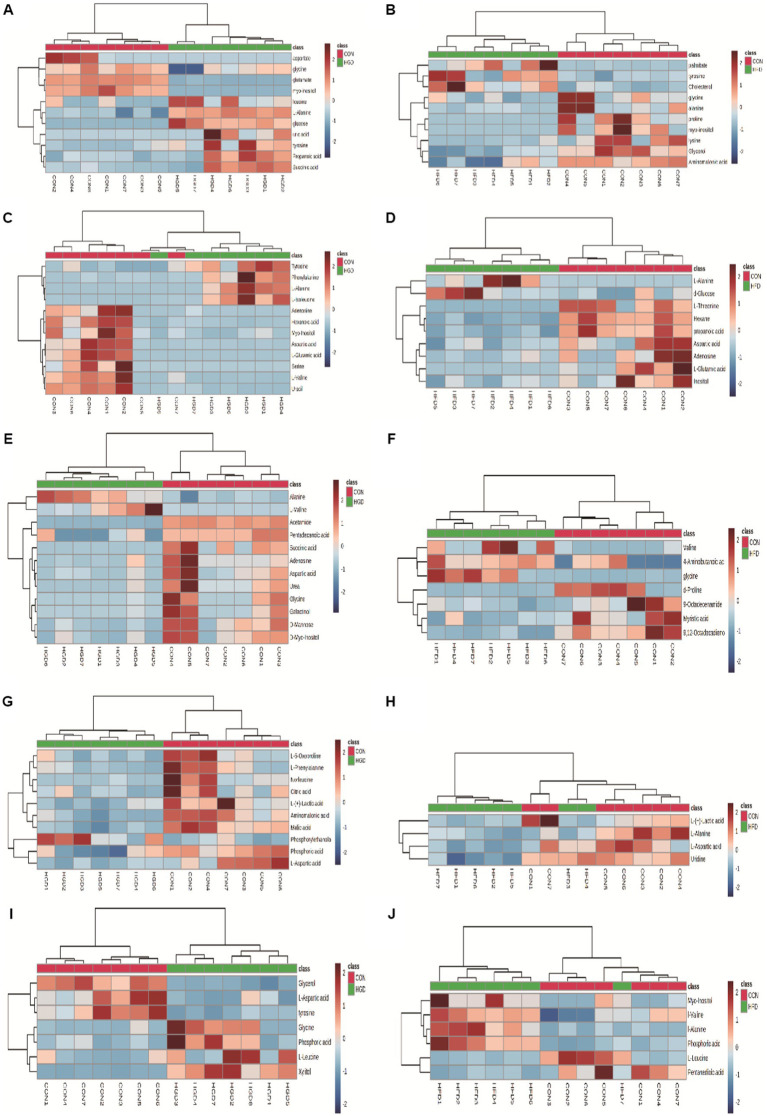
Heatmaps of metabolite expression differences between HGD and HFD groups: serum (**A**: HGD, **B**: HFD), heart (**C**: HGD, **D**: HFD), liver (**E**: HGD, **F**: HFD), brain (**G**: HGD, **H**: HFD), kidney (**I**: HGD, **J**: HFD). The shades of red and blue signify upregulation and downregulation; the darker the color, the greater the shift. Samples and metabolites are represented by columns and rows, respectively.

Analyzing the data for the discovered metabolites, we additionally used heatmaps (MetaboAnalyst v5.0) to identify two separate clusters with low overlap for most metabolites in two diet groups (HGD groups: Figures 2A,C,E,G,I; HFD groups: Figures 2B,D,F,H,J).

### Analysis of metabolic pathways

**Figure 3:**
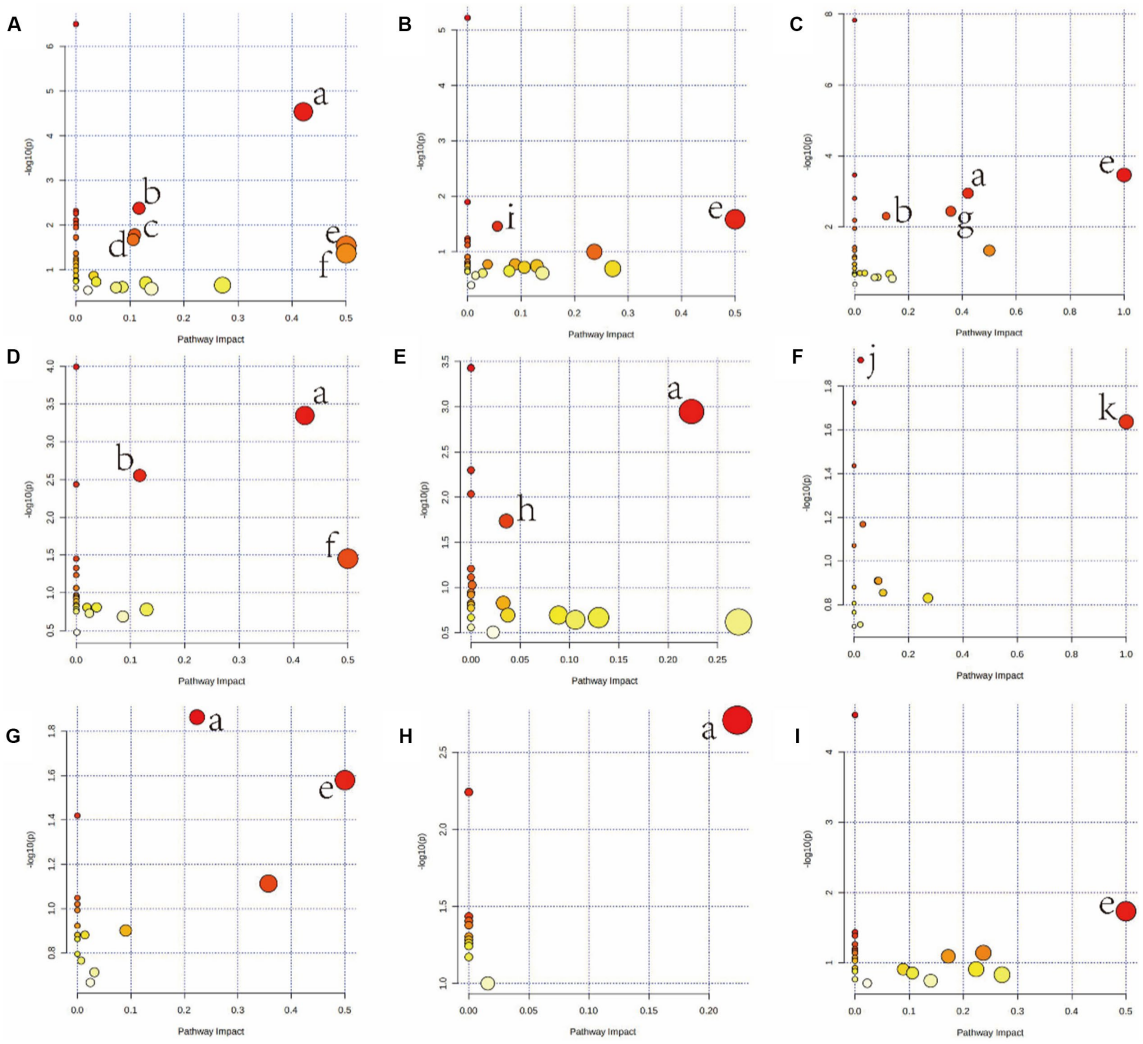
Summary of pathway analysis using MetaboAnalyst v5.0. Serum (**A**: HGD, **B**: HFD); heart (**C**: HGD, **D**: HFD); liver (**E**: HGD, **F**: HFD); brain (**G**: HGD, **H**: HFD); kidney (**I**: HGD). **(a)** Alanine, aspartate, and glutamate metabolism. **(b)** Arginine biosynthesis. **(c)** Glutathione metabolism. **(d)** Glyoxylate and dicarboxylate metabolism. **(e)** Phenylalanine, tyrosine, and tryptophan biosynthesis. **(f)** D-Glutamine and D-glutamate metabolism. **(g)** Phenylalanine metabolism. **(h)** Galactose metabolism. **(i)** Primary bile acid biosynthesis. **(j)** Arginine and proline metabolism. **(k)** Linoleic acid metabolism.

**Figure 4:**
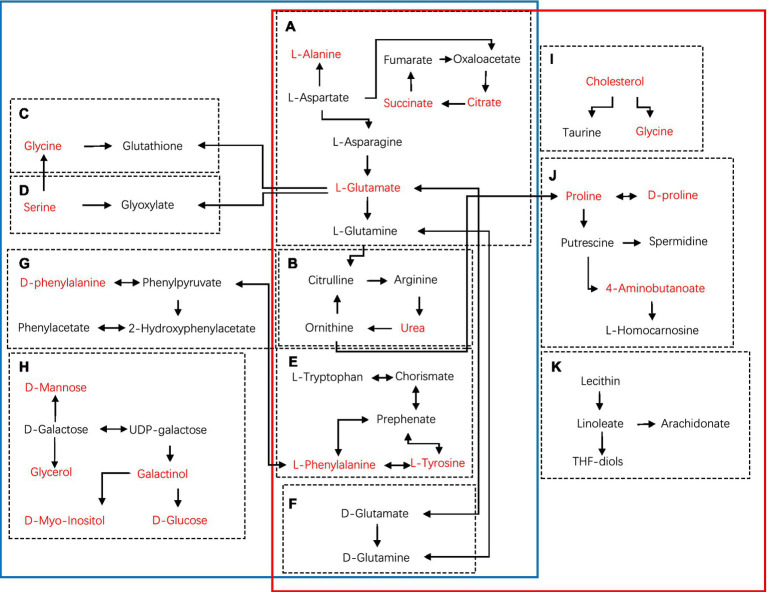
Schematic diagram of metabolic pathways using KEGG in main tissues (serum, heart, liver, brain, kidney) of HGD and HFD compared to the controls (blue solid line: HGD group; red solid line: HFD group). **(A)** Alanine, aspartate, and glutamate metabolism. **(B)** Arginine biosynthesis. **(C)** Glutathione metabolism. **(D)** Glyoxylate and dicarboxylate metabolism. **(E)** Phenylalanine, tyrosine, and tryptophan biosynthesis. **(F)** D-Glutamine and D-glutamate metabolism. **(G)** Phenylalanine metabolism. **(H)** Galactose metabolism. **(I)** Primary bile acid biosynthesis. **(J)** Arginine and proline metabolism. **(K)** Linoleic acid metabolism. Metabolites marked in red represent the significant biomarkers found in main tissues.

Furthermore, metabolites were examined using MetaboAnalyst v5.0^2^ and KEGG database (see footnote 1) to investigate the metabolic pathways among diets and the control group. The following are some important pathways (raw *p* < 0.05, impact >0; Table 3): glyoxylate and dicarboxylate metabolism; D-glutamine and D-glutamate metabolism; arginine biosynthesis; Alanine, aspartate, and glutamate metabolism; phenylalanine, tyrosine, and tryptophan biosynthesis; glutathione metabolism; phenylalanine metabolism; and galactose metabolism are all included in the HGD group (Figures 3A,C,E,G,I). Moreover, biosynthesis of phenylalanine, tyrosine, and tryptophan; main bile acid biosynthesis; arginine biosynthesis; D-glutamine and D-glutamate metabolism; arginine and proline metabolism; alanine, aspartate, and glutamate metabolism; and linoleic acid metabolism were all studied in the HFD group (Figures 3B,D,F,H), but no data of kidney meet the screening criteria in HFD group. A summary of metabolites and metabolic pathways is shown in Figure 4.

**Table 3:** Pathway analysis by MetaboAnalyst 5.0.

	HGD			HFD		
Tissue	Pathway	Raw p	Impact	Pathway	Raw p	Impact
Serum	Alanine, aspartate and glutamate metabolism	2.89E-05	0.42	Phenylalanine, tyrosine and tryptophan biosynthesis	2.63E-02	0.50
	Arginine biosynthesis	4.21E-03	0.12	Primary bile acid biosynthesis	3.51E-02	0.06
	Glutathione metabolism	1.65E-02	0.11			
	Glyoxylate and dicarboxylate metabolism	2.14E-02	0.11			
	Phenylalanine, tyrosine and tryptophan biosynthesis	2.89E-02	0.50			
	D-Glutamine and D-glutamate metabolism	4.31E-02	0.50			
Heart	Phenylalanine, tyrosine and tryptophan biosynthesis	3.46E-04	1.00	Alanine, aspartate and glutamate metabolism	4.49E-04	0.42
	Alanine, aspartate and glutamate metabolism	1.13E-03	0.42	Arginine biosynthesis	2.78E-03	0.12
	Phenylalanine metabolism	3.68E-03	0.36	D-Glutamine and D-glutamate metabolism	3.54E-02	0.50
	Arginine biosynthesis	5.02E-03	0.12			
Liver	Alanine, aspartate and glutamate metabolism	1.13E-03	0.22	Arginine and proline metabolism	1.20E-02	0.02
	Galactose metabolism	1.83E-02	0.04	Linoleic acid metabolism	2.31E-02	1.00
Brain	Alanine, aspartate and glutamate metabolism	1.37E-02	0.22	Alanine, aspartate and glutamate metabolism	1.96E-03	0.22
	Phenylalanine, tyrosine and tryptophan biosynthesis	2.63E-02	0.50			
Kidney	Phenylalanine, tyrosine and tryptophan biosynthesis	1.85E-02	0.50			

## Discussion

Pathophysiology and metabolic disturbances are produced by glucolipid metabolic diseases. For excess models and nutritional deficiencies, high-throughput metabolomics data is becoming accessible, which is important for understanding the complicated nutritional interactions of complete organisms (8). As a result, we conducted primary tissue metabolite profiling in mice and observed alterations in a few metabolites. Long-term use of HGD and HFD resulted in a weight increase in the current research. The progressive accumulation of body fat was the major cause of weight increase. Furthermore, the HGD and HFD groups showed severe metabolic impairments, including increased glucose, TG, and TC blood levels, indicating that HGD and HFD induced obesity and disrupted glucose homeostasis, which is consistent with earlier results (12).

We compared the differences in serum and main tissue metabolite levels between the two divergent dietary interventions. In both HGD and HFD, the majority of the metabolites whose concentrations altered were associated with AA, lipids, and energy metabolism (Table 2; Figure 3). Additional support for the impact of nutrition on the metabolome was found through the discovery of specific metabolites, such as the amino acids L-valine, L-isoleucine, L-leucine, phenylalanine, and L-threonine, as well as phenylalanine and serine. Metabolic pathways significantly associated with HGD and HFD were consistent with altered dietary patterns, including amino acid metabolism and lipid or fatty acid-related metabolic pathways (Table 3; Figure 4). In these pathways, there are several same pathways in both diet groups, included alanine, aspartate, and glutamate metabolism, arginine biosynthesis, phenylalanine, tyrosine, and tryptophan biosynthesis, and D-Glutamine and D-glutamate metabolism. And there are three pathways in each diet group, glutathione metabolism, phenylalanine metabolism, galactose metabolism in HGD group, and primary bile acid biosynthesis, arginine and proline metabolism, linoleic acid metabolism in HFD group. Therefore, in light of the distinct metabolic pathways observed in each group, we can select the most suitable biomarker for modulating glycolipid metabolism.

Obesity has been highly related to detrimental glucose profiles, and leads to the development of type 2 diabetes (T2DM) (13). In this research, both HGD and HFD were linked to insulin dysfunction, with the high-energy diets promoting increased insulin resistance and raised blood glucose levels. Consequently, the L-valine, L-leucine, L-isoleucine, tyrosine, phenylalanine, and serine levels underwent significant alternation in the two diet groups of our study. Increase in branch-chain amino acids (BCAAs) and phenylalanine may attenuate insulin sensitivity and enhance insulin resistance (14). Researchers have studied amino acids and related metabolites in normoglycemic individuals and suggest that aromatic and BCAAs could be predictors of diabetes (15). The branched-chain amino acids, L-valine and L-leucine, also have important roles in inflammation and energy metabolism (16, 17). Accumulating evidence supports the theory that a high-energy diet, metabolic imbalance, and inflammation interact in states of over-nutrition (12, 18). Further research confirmed that consumption of HGD and HFD seems to be linked to unfavorable alterations in metabolic profiles that are associated with inflammation (19, 20).

HGD seemed have a greater impact on murine metabolism than HFD. Our study noted that HGD increased the diversity of metabolites in mice. Glucogenic (valine, glycine, serine, alanine, proline, and asparagine) and ketogenic (isoleucine, tyrosine, leucine, and phenylalanine) amino acids have substantially different related metabolites. Feeding mice a high-energy diet induces tissue-specific changes that ultimately result in the onset of metabolic disorders. For example, the heart is a “metabolic omnivore” and can utilize various fuel sources including fats, sugars, ketone bodies, lactate, and amino acids (21). Ketogenic amino acids can be utilized by the liver to form ketone bodies, which are then transported to other organs (22). Unlike the muscle and liver, brain tissue obtain energy from both ketones and glucose. Moreover, glycogenolysis and gluconeogenesis occur in the liver as well as in renal proximal tubules to maintain a steady energy supply to the brain (23).

Aromatic amino acids have a close relationship with body metabolism and can have a significant impact on liver function. Studies have shown that changes in plasma aromatic amino acids can be indicative of atypical liver function and enhanced protein catabolism (24). These changes are often associated with various health conditions, such as liver diseases, metabolic disorders, and cancer, and can be used in clinical diagnosis and treatment. In our investigation, oxidative stress-related metabolites including L-glutamic acid and serine were dramatically altered. A high-glucose diet has been shown to enhance oxidative stress in the livers of mice, potentially leading to diabetes and metabolic syndrome (25). Furthermore, in other disease models, elevated tyrosine, proline, glycine, and alanine are thought as indicators of mitochondrial malfunction (26).

Obesity is a non-communicable condition characterized by the buildup of excessive fat. Significantly, obesity and changes in the gut flora and metabolites are readily caused by a high-fat diet (27, 28). Glucose was the primary source of energy for HGD-fed mice, but lipids were utilized more often in the HFD group. HFD also disrupts normal cellular metabolic programming and perturbs the activities of the regulators of nutrient homeostasis, including mTOR, AMPK, and CREB, which contribute to metabolic diseases (29–31). Our study has demonstrated that the lipid metabolism of blood, cardiac muscle, and liver is susceptible to the effects of HFD, resulting in alterations of lipid levels in these tissues. When mice were given high-fat diet, lipid metabolism was found to be greatly elevated, and genes linked to the production and breakdown of fat in the liver were shown to be significantly increased (32). In our study, for mice in HFD, most of the metabolites in tissues were related to lipid metabolism, such as glycerol, cholesterol, palmitate, myristic acid, propanoic acid, 9,12-Octadecadienoic acid, and myo-inositol, as well as few AAs. The saturated fatty acid palmitate can induce a mixed inflammatory response by upregulating inflammatory and endoplasmic reticulum (ER) stress genes, and increasing the expression of appetite-stimulating NPY (neuropeptide Y) neurons leading to difficulty in weight management (33). 9,12-octadecadienoic acid is able to reduce the risk of cardiovascular disease and exert a certain protection on the cardiovascular system (34). Myristic acid also produces a significant increase in systemic inflammatory response syndrome and shows potential as a biomarker in the diagnosis of septic patients (35). Moreover, myristic acid improves hyperglycemia and reduces body weight, and could be used in the treatment of diabetes (36).

In addition, long-term consumption of HFD affects brain health, decreases hippocampus volume, and impairs cognitive function (37, 38). In our study, HFD supplied alanine, glycine, and 4-aminobutanoic acid, which exert negative effects on the brain. This may explain the association between high fat intakes and increased incidence of depression and anxiety in individuals with obesity.

Finally, our findings show that whereas both HGD and HFD impacted tissue metabolomes, they exhibited different metabolic signatures. The dietary intervention had a significant impact on tissue metabolomes in mice, indicating that metabolomics might be a useful tool for detecting illness development in animal models. However, our research is not without limitations, and there is still much more to be explored in this field. In the next phase of our study, we plan to include additional groups, such as those following a high-fructose diet and a high-fiber diet, to further investigate the effects of different dietary components on the body’s metabolism. Furthermore, we will concentrate more on the influence of varying nutrient consumption quantities on an individual’s metabolic health.

## Data availability statement

The raw data supporting the conclusions of this article will be made available by the authors, without undue reservation.

## Ethics statement

The animal study was reviewed and approved by Jining Medical University's ethics committee (Protocol #JNMC2020DWRM0076).

## Author contributions

DX, YGu, and PJ designed the study. SZ, CG, YL, RY, YGa, and HL performed the experiments. DX, YZ, and XX analyzed the data. DX, YZ, ZR, and PJ wrote the manuscript. All authors contributed to the article and approved the submitted version.

## Funding

This work was supported by Bethune Charitable Foundation [No: B-19-H-20200622], Scientific Research Foundation of Shandong Medical Association [No: YXH2020ZX053], Natural Science Foundation of Shandong Province [No: ZR2020MH375], and Foundation of Xuzhou Medical University [No: XYFY202213].

## Conflict of interest

The authors declare that the research was conducted in the absence of any commercial or financial relationships that could be construed as a potential conflict of interest.

## Publisher’s note

All claims expressed in this article are solely those of the authors and do not necessarily represent those of their affiliated organizations, or those of the publisher, the editors and the reviewers. Any product that may be evaluated in this article, or claim that may be made by its manufacturer, is not guaranteed or endorsed by the publisher.

## Footnotes

^1^ http://www.kegg.jp

^2^ http://www.metaboanalyst.ca
