# Supplementary material for: The impact of high-glucose or high-fat diets on the metabolomic profiling of mice
Source: Front Nutr. 2023 Jul 10;10:1171806. doi: 10.3389/fnut.2023.1171806 (PMC10363684; doi:10.3389/fnut.2023.1171806)
Supplement: Supplementary file 1 [file Data_Sheet_1.PDF]

| Ingredient | RD20050902 | | RD20050903 | |
| --- | --- | --- | --- | --- |
| | g/kg | kcal | g/kg | kcal |
| Casein | 140 | 560 | 140 | 560 |
| L-Cystine | 1.8 | 7.2 | 1.8 | 7.2 |
| Corn Starch | 15.692 | 62.768 | 10 | 40 |
| Maltodextrin | 125 | 500 | 125 | 500 |
| Fructose | 0 | 0 | 0 | 0 |
| Glucose | 480 | 1920 | 0 | 0 |
| Sucrose | 100 | 400 | 100 | 400 |
| Cellulose | 50 | 0 | 50 | 0 |
| Soybean Oil | 40 | 360 | 40 | 360 |
| Lard | 0 | 0 | 216 | 1944 |
| t-Butyhydroquinone | 0.008 | 0 | 0.008 | 0 |
| Mineral Mix S10022 | 35 | 0 | 35 | 0 |
| Vitamine Mix V1003 | 10 | 40 | 10 | 40 |
| Choline Bitartrate | 2.5 | 0 | 2.5 | 0 |
| Total | 1000 | 3850 | 730.308 | 3850 |

D10012M (AIN-93M)

| g | kcal |
|---|---|
| 140 | 560 |
| 1.8 | 7.2 |
| 495.692 | 1982.768 |
| 125 | 500 |
| 0 | 0 |
| 0 | 0 |
| 100 | 400 |
| 50 | 0 |
| 40 | 360 |
| 0 | 0 |
| 0.008 | 0 |
| 35 | 0 |
| 10 | 40 |
| 2.5 | 0 |
| 1000 | 3850 |
