# Supplementary figures and images for: The impact of high-glucose or high-fat diets on the metabolomic profiling of mice
Source: Front Nutr. 2023 Jul 10;10:1171806. doi: 10.3389/fnut.2023.1171806 (PMC10363684; doi:10.3389/fnut.2023.1171806)

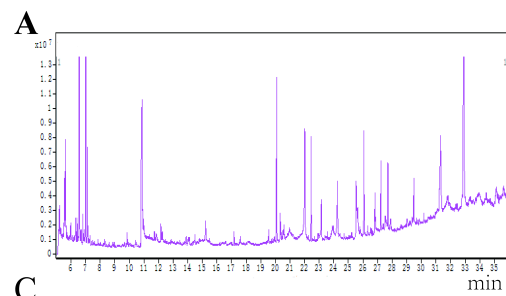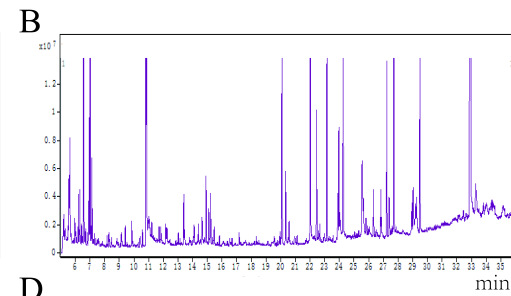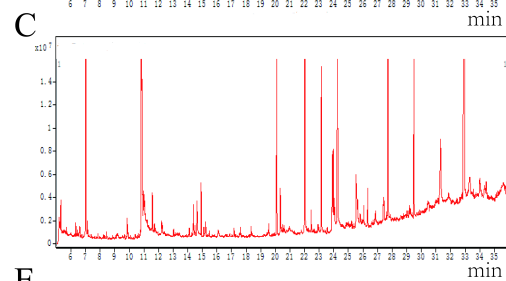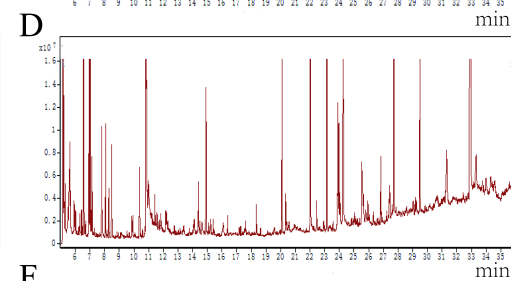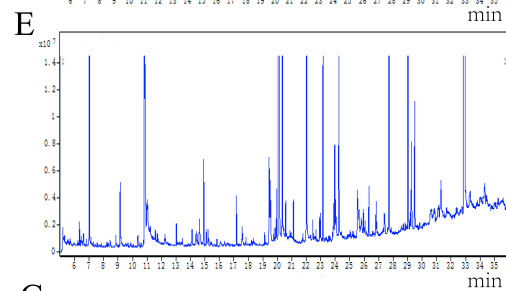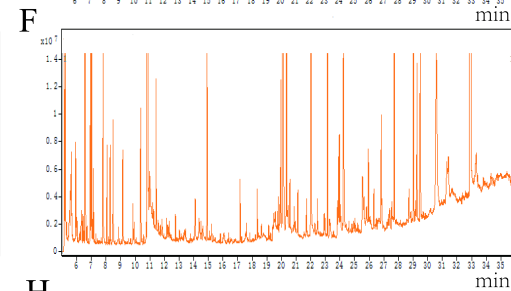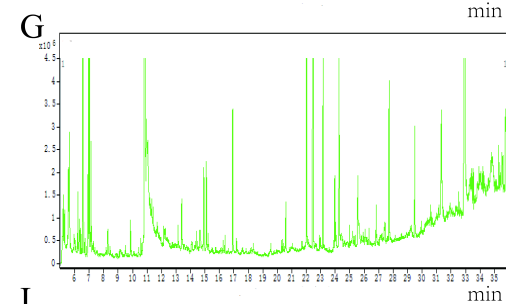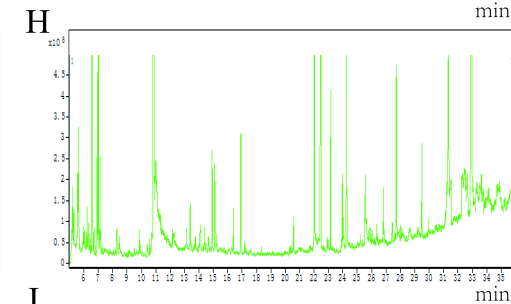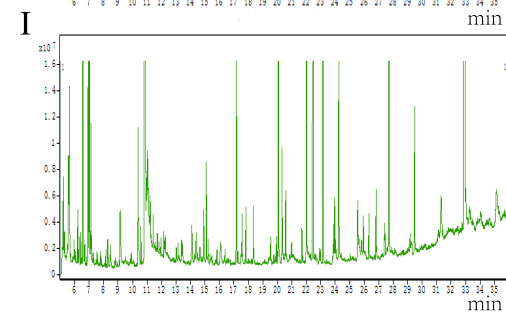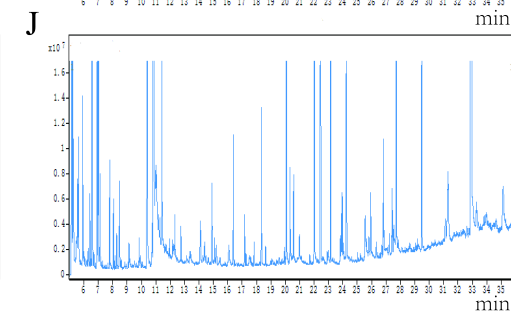

Supplement: Supplementary file 2 [file Data_Sheet_2.PDF]
